# Functional crosstalk between the mitochondrial PTP and K_ATP_ channels determine arrhythmic vulnerability to oxidative stress

**DOI:** 10.3389/fphys.2014.00264

**Published:** 2014-07-16

**Authors:** Chaoqin Xie, Justin Kauffman, Fadi G. Akar

**Affiliations:** Department of Medicine, Cardiovascular Institute, Mount Sinai School of MedicineNew York, NY, USA

**Keywords:** mitochondria, oxidative stress, permeability transition pore, mitochondrial K_ATP_ channel, arrhythmia

## Abstract

**Background:** Mitochondrial permeability transition pore (mPTP) opening is a terminal event leading to mitochondrial dysfunction and cell death under conditions of oxidative stress (OS). However, mPTP blockade with cyclosporine A (CsA) has shown variable efficacy in limiting post-ischemic dysfunction and arrhythmias. We hypothesized that strong feedback between energy dissipating (mPTP) and cardioprotective (mK_ATP_) channels determine vulnerability to OS.

**Methods and Results:** Guinea pig hearts (*N* = 61) were challenged with H_2_O_2_ (200 μM) to elicit mitochondrial membrane potential (ΔΨ_m_) depolarization. High-resolution optical mapping was used to measure ΔΨ_m_ or action potentials (AP) across the intact heart. Hearts were treated with CsA (0.1 μM) under conditions that altered the activity of mK_ATP_ channels either directly or indirectly via its regulation by protein kinase C. mPTP blockade with CsA markedly blunted (*P* < 0.01) OS-induced ΔΨ_m_ depolarization and delayed loss of LV pressure (LVP), but did not affect arrhythmia propensity. Surprisingly, prevention of mK_ATP_ activation with the chemical phosphatase BDM reversed the protective effect of CsA, paradoxically exacerbating OS-induced ΔΨ_m_ depolarization and accelerating arrhythmia onset in CsA treated compared to untreated hearts (*P* < 0.05). To elucidate the putative molecular mechanisms, mPTP inhibition by CsA was tested during conditions of selective PKC inhibition or direct mK_ATP_ channel activation or blockade. Similar to BDM, the specific PKC inhibitor, CHE (10 μM) did not alter OS-induced ΔΨ_m_ depolarization directly. However, it completely abrogated CsA-mediated protection against OS. Direct pharmacological blockade of mK_ATP_, a mitochondrial target of PKC signaling, equally abolished the protective effect of CsA on ΔΨ_m_ depolarization, whereas channel activation with 30 μM Diazoxide protected against ΔΨ_m_ depolarization (*P* < 0.0001). Conditions that prevented mK_ATP_ activation either directly or indirectly via PKC inhibition led to accelerated ΔΨ_m_ depolarization and early onset of VF in response to OS. Investigation of the electrophysiological substrate revealed accelerated APD shortening in response to OS in arrhythmia-prone hearts.

**Conclusions:** Cardioprotection by CsA requires mK_ATP_ channel activation through a PKC-dependent pathway. Increasing mK_ATP_ activity during CsA administration is required for limiting OS-induced electrical dysfunction.

## Introduction

Mitochondria are central mediators of the cardiac response to oxidative stress (OS), as they respond to reactive oxidative species (ROS) through a host of ROS sensitive channels, which can either amplify or limit ROS-induced injury (O'Rourke et al., 2007). Of key importance to OS-induced mitochondrial dysfunction are the inner membrane anion channel (IMAC) and components of the mitochondrial permeability transition pore (mPTP). Both channel complexes activate in response to rising ROS levels. However, as described by Aon and colleagues, they exhibit a hierarchal activation pattern (Aon et al., 2007): IMAC activates first in response to moderate levels of OS followed by the activation of the large conductance mPTP, which leads to irreversible mitochondrial membrane potential (ΔΨ_m_) depolarization (i.e., induction of the mitochondrial permeability transition, MPT) (Aon et al., 2007). Indeed, both channels have been implicated in mitochondrial dysfunction through a regenerative, autocatalytic process known as ROS-induced ROS-release (RIRR) which can culminate in electrical dysfunction or cell death (Zorov et al., 2000, 2006; Yang et al., 2010; Biary et al., 2011; Akar, 2013).

While the role of the mPTP in the activation of necrotic cell death pathways is well established, we and others have demonstrated the importance of IMAC in OS-induced arrhythmias (Akar et al., 2005; Akar and O'Rourke, 2011). In those studies, IMAC (but not mPTP) blockade effectively abrogated pathological OS-induced ΔΨ_m_ and action potential (AP) oscillations and prevented post-ischemic arrhythmias (Akar et al., 2005). It is important to note, however, that our previous studies focused on relatively short episodes of ischemia-reperfusion (I/R) injury which did not result in myocardial infarction (MI) (Akar et al., 2005; Lyon et al., 2010). Given the hierarchal nature of mitochondrial channel activation (Aon et al., 2007), we hypothesized that the mPTP may only play a prominent role under conditions of more extreme OS. Indeed, the immunosuppressive agent, Cyclosporin A (CsA), a desensitizer of the mPTP in the heart through its effect on Cyclophilin-D (CyP-D), has been shown to be effective in reducing infarct size in patients (Piot et al., 2008; Hausenloy et al., 2012). Despite these encouraging clinical findings, the efficacy of CsA in preventing arrhythmias is unclear (Arteaga et al., 1992; Ko et al., 1997; Schreiner et al., 2004), and recent experimental, preclinical (Lie et al., 2008), and clinical findings (Ghaffari et al., 2013) have cast new doubts regarding the overall utility and safety profile of CsA.

Mitochondria play a dual role: on the one hand, they initiate cell death and injury pathways through energy dissipating channels, such as the mPTP, but on the other, they act as central mediators of cardioprotection (Penna et al., 2013). Indeed, multiple stimuli (i.e., ischemic pre- and post-conditioning protocols, pharmacological agents and volatile anesthetics) limit cardiac damage by activating powerful cardioprotective signaling cascades which converge on mitochondria, in large part, through mitochondrial ATP-sensitive K (mK_ATP_) channels (Liu et al., 1998, 1999; Sato and Marban, 2000; Garlid et al., 2009). Whether mK_ATP_ channels functionally interact with components of the mPTP in a manner that modulates the response of the heart to OS is unclear. In the present study, we set out to address this issue directly in a model of acute OS that was specifically designed to elicit significant ΔΨ_m_ depolarization and electrical dysfunction. We found that the efficacy of CsA in limiting OS-induced mitochondrial and electrical dysfunction was dictated by strong functional cross-talk between the mPTP and mK_ATP_ channels through a protein kinase C (PKC)-dependent pathway. Our findings highlight the importance of enhancing mK_ATP_ channel activity during CsA administration for limiting OS-induced electrical dysfunction, and may explain discrepant reports of the utility and potential toxicity of CsA.

## Materials and methods

All procedures involving the handling of animals were approved by the Animal Care and Use Committee of the Mount Sinai School of Medicine and adhered with the Guide for the Care and Use of Laboratory Animals published by the *National Institutes of Health*. Guinea pig hearts (*N* = 61) were rapidly excised, washed with ice cold cardioplegic solution, transferred to a Langendorff apparatus, and retrogradely perfused through the aorta with oxygenized (95% O_2_–5% CO_2_) Tyrodes solution containing (in mM): 130 NaCl, 1.2 MgSO_4_, 25 NaHCO_3_, 4.75 KCl, 5 Dextrose, and 1.25 CaCl_2_ at 36 ± 1°C. Perfusion pressure was maintained at 60–65 mmHg by adjusting perfusion flow rate. Hearts were suspended in the buffer filled, temperature controlled chamber, as we have recently reported (Jin et al., 2010; Lyon et al., 2010). Volume-conducted electrocardiograms were recorded for rhythm analysis using non-contact silver electrodes placed within the chamber. ECG signals were recorded continuously throughout the entire *ex vivo* perfusion protocol. Left ventricular (LV) cavity pressure (LVP) was measured using a buffer filled latex balloon (Harvard apparatus) that was carefully inserted through the mitral valve into the LV cavity. Signals were amplified (ECG100-MP150 Amplifier, Biopac Systems, CA, USA) and displayed in real-time using the *AcqKnowledge 3.9* software package (Biopac Systems). Hearts were positioned such that the mapping field was centered over a 4 × 4-mm^2^ region of LV epicardium, midway between apex and base. These preparations remain stable for over 4 h of perfusion.

### High-resolution optical ΔΨ_m_ imaging in *ex vivo* perfused guinea pig hearts

We used a validated semi-quantitative imaging technique of optical ΔΨ_m_ mapping using the ΔΨ_m_-sensitive dye tetramethylrhodamine methylester (TMRM) (Jin et al., 2010; Lyon et al., 2010; Smeele et al., 2011; Nederlof et al., 2013). This method allows the assessment of mitochondrial function at a subcellular resolution within the intact organ (Jin et al., 2010; Lyon et al., 2010). Briefly, following cannulation, hearts were allowed to stabilize for 20 min at physiological temperature. Hearts were then stained with TMRM (250 nM; Molecular Probes Inc.) mixed in a 500 mL volume of Tyrodes solution (dye loading phase) for 20 min. This was followed by a 20–30 min dye washout phase. TMRM background fluorescence intensity was measured periodically (in 1 min intervals) throughout the entire experiment using a 6400 pixel CCD based optical imaging approach that allowed the measurement of normalized ΔΨ_m_ with subcellular resolution (50 μm) over a 4 × 4-mm^2^ window of the epicardial surface. To measure TMRM background fluorescence, hearts were excited with filtered light (525 ± 20 nm) emitted from a quartz tungsten halogen lamp (Newport Corporation, CT, USA). Emitted fluorescence was filtered (585 ± 20 nm for TMRM) and focused onto a high-resolution CCD camera (Scimeasure, GA, USA). During dye washout, the stability of TMRM background fluorescence was evaluated in real-time, as this baseline level served for normalization purposes during OS. In all experiments, the dye washout phase was associated with stable signal intensity.

High-throughput analysis of optical signals was performed using custom designed software. Peak emitted TMRM fluorescence signal from each of 6400 pixels was measured before and after excitation. TMRM background fluorescence was baseline corrected by subtracting fluorescence levels before dye staining (<0.1%) for each pixel. Background corrected TMRM fluorescence (ΔΨ_m_) during the OS protocol was then normalized to the value of steady-state TMRM fluorescence achieved during the dye washout phase for each of the 6400 individual pixels. Normalized ΔΨ_m_ measurements during OS across the imaged 4 × 4-mm^2^ region of the heart were plotted as contour maps using *Delta Graph 5.6* (Red Rock Software).

### High-resolution optical action potential mapping in *ex vivo* perfused hearts

For optical AP mapping studies, hearts were stained with di-4-ANEPPS for 10 min as previously described (Akar et al., 2005; Xie et al., 2013). Hearts were paced at a steady-state pacing cycle length (PCL) of 300 ms. Unlike ΔΨ_m_ imaging, optical AP mapping requires motion suppression; hence 10 mM BDM was used in this subset of studies.

### *Ex vivo* models of acute OS leading to irreversible ΔΨ_m_ depolarization

**Figure 1:**
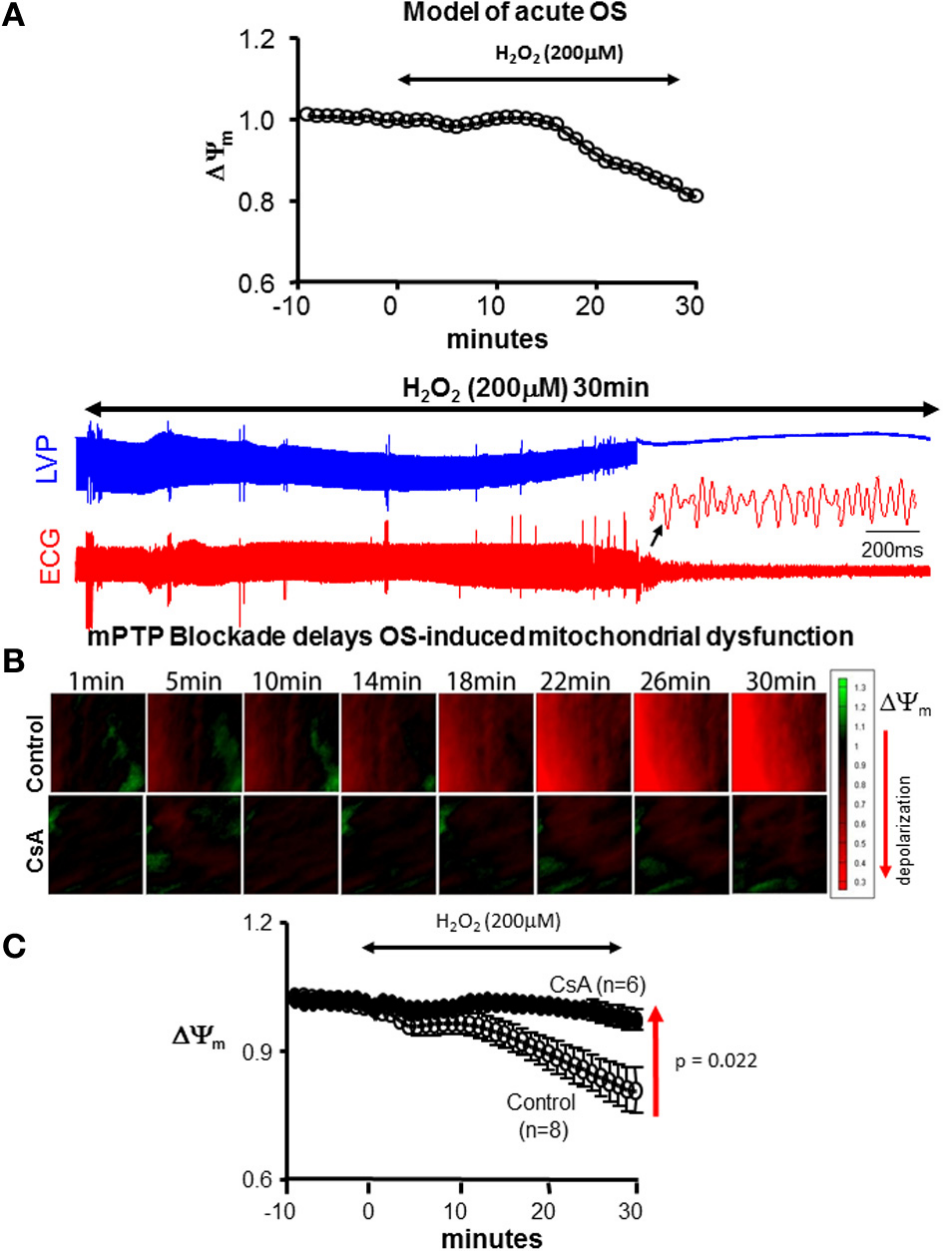
**CsA delays OS-induced mitochondrial and contractile dysfunction. (A)** Model of acute OS. Continuous 200 μM H_2_O_2_ perfusion for 30 min leads to ΔΨ_m_ depolarization and initiation of VF. **(B)** Sequences of isopotential contour maps depicting the spatio-temporal distribution of ΔΨ_m_ in representative control (untreated) and CsA-treated hearts following challenge with H_2_O_2_ (200 μM) for 30 min. **(C)** Average ΔΨ_m_ response to acute OS in all control and CsA treated hearts.

Perfusion of hearts with H_2_O_2_ is a well-established model of acute OS that results in triggered activity (Sato et al., 2009) as well as sustained atrial (Morita et al., 2010) and ventricular tachyarrhythmias (Morita et al., 2009; Biary et al., 2011). Following dye washout and stabilization, hearts were perfused with 200 μM H_2_O_2_ (Sigma-Aldrich) in Tyrodes for 30 min to elicit OS. We found that this model consistently gives rise to the regenerative process of RIRR (Biary et al., 2011), which culminates in significant ΔΨ_m_ depolarization, contractile and electrical dysfunction (Figure 1). This model served as the platform for investigating the role of mitochondrial ion channel complexes in the modulation of OS-induced ΔΨ_m_ and arrhythmias. Specifically, we focused on the role of the mPTP and its cross-talk with the mK_ATP_. The following agents and concentrations were used in the present study: (a) CsA (0.1 μM, mPTP blocker), (b) Chelerythrine Chloride (CHE, 10 μM, PKC inhibitor), (c) 5-Hydroxydecanoate (5-HD, 100 μM, mK_ATP_ blocker), and (d) diazoxide (DZX, 30μM, mK_ATP_ agonist). Drug delivery was initiated 10 min before OS and was maintained throughout the entire protocol.

### Statistical analysis

Values were expressed as mean ± SE. Differences between two groups were compared using the Student's *t*-test and were considered significant for *p* < 0.05.

## Results

### Acute model of OS-induced mitochondrial and electrical dysfunction

The main objective of the present work was to test the efficacy of mPTP blockade in protecting against OS-induced mitochondrial and electrical dysfunction. To that end, we used a simple *ex vivo* model of acute OS by H_2_O_2_ challenge (Figure 1A). This consistently resulted in significant ΔΨ_m_ depolarization, contractile, and electrical dysfunction. Within 40 min of H_2_O_2_, 5/6 hearts underwent spontaneous onset of VF with the remaining heart exhibiting electrical silence. As such, this model served as a reliable platform for investigating the role of mitochondrial ion channel complexes in the functional modulation of OS-induced mitochondrial and electrical dysfunction.

### CsA protects against OS-induced mitochondrial and contractile but not electrical dysfunction

We began by investigating the efficacy of CsA in altering the functional response of hearts to acute OS. Shown in Figure 1B are ΔΨ_m_ isopotential contour maps from untreated (control) and CsA-treated hearts following challenge with H_2_O_2_. Also shown are the average normalized ΔΨ_m_ responses from all hearts. As expected, H_2_O_2_ challenge resulted in significant ΔΨ_m_ depolarization in control hearts. On average, ΔΨ_m_ was reduced by 22.1% within 30 min of H_2_O_2_ perfusion. Remarkably, CsA treatment completely abolished this response, as ΔΨ_m_ remained fully polarized during the same time-course in CsA-treated compared to untreated control hearts (Figure 1). Following 30 min of H_2_O_2_ challenge, ΔΨ_m_ was 19.3% greater (*p* = 0.021) in CsA-treated compared to control hearts.

**Figure 2:**
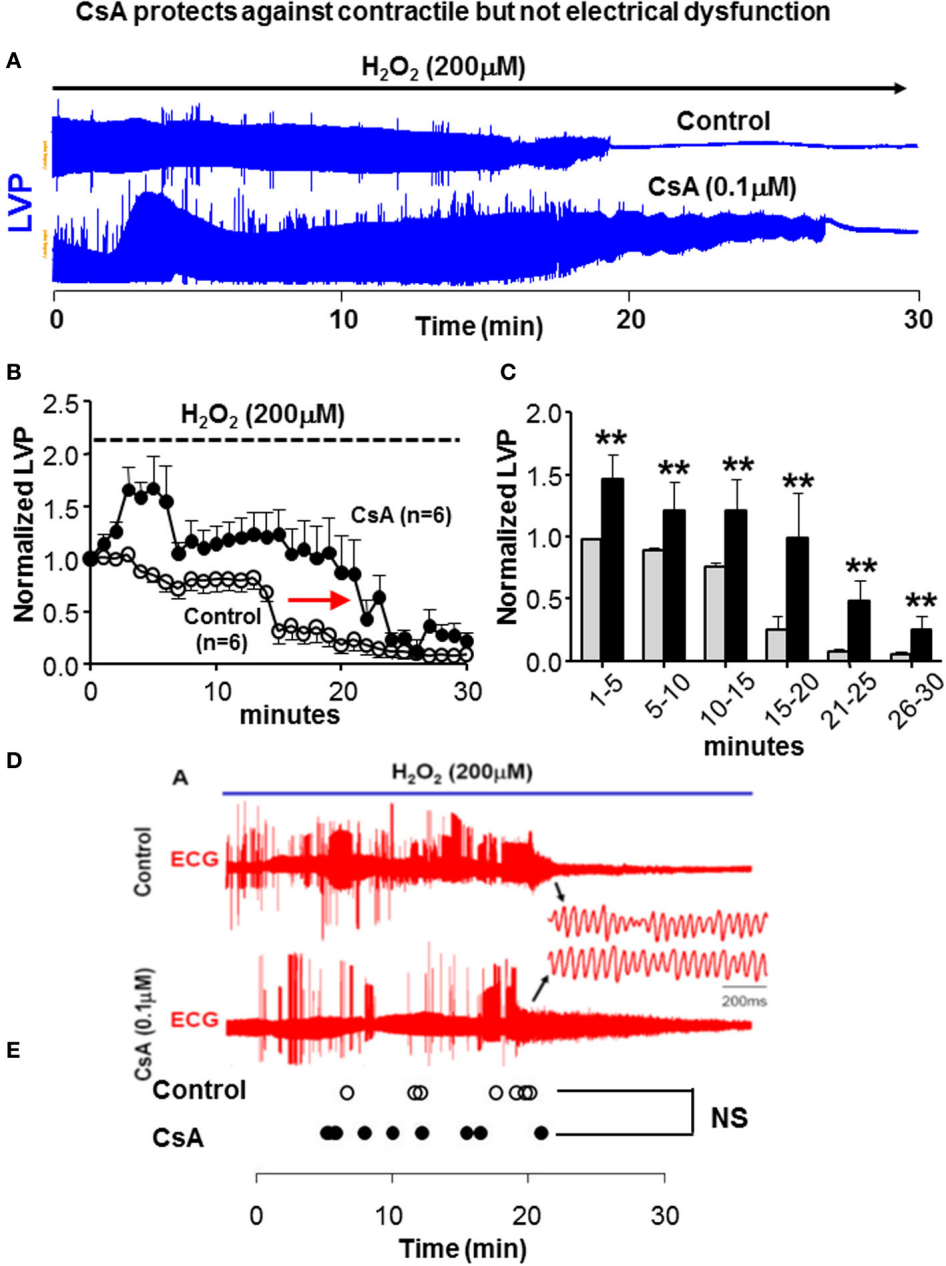
**CsA does not protect against electrical dysfunction. (A)** Representative time-series of LV pressure waveforms during 30 min of H_2_O_2_ challenge, indicating reduction and ultimate loss of contractile function. Loss of LVP is delayed in CsA treated hearts. **(B,C)** Average LVP normalized to the baseline pre-H_2_O_2_ value in all control and CsA hearts. Experiments summarized in **(A–C)** where performed under BDM-free conditions. **(D,E)** ECG traces in control and CsA treated hearts during H_2_O_2_ challenge and time of onset of VF in all hearts irrespective of BDM presence in the purfase. The transition to VF is not delayed by CsA treatment, indicating lack of electrical protection by CsA. BDM, 2,3-Butanedione monoxime; LVP, Left ventricular pressure; ECG, electrocardiogram; VF, ventricular fibrillation. ^**^p < 0.01.

We next tested whether modulation of OS-induced ΔΨ_m_ depolarization and its prevention by CsA had a functional impact in terms of contractile (Figures 2A–C) and electrical (Figures 2D,E) properties. As expected, H_2_O_2_ perfusion in control hearts resulted in a gradual decrease and eventual loss of contractile function. Interestingly, prevention of ΔΨ_m_ depolarization by CsA was associated with relative protection against contractile dysfunction as the loss of LVP was delayed by >8 min, *p* = 0.01 (Figure 2). We next investigated whether protection against OS-induced mitochondrial dysfunction by CsA translated into an electrical benefit by either preventing or delaying the onset of VF. Surprisingly, we found that CsA treatment failed to protect against the incidence of arrhythmias as the time to onset of VF following H_2_O_2_ challenge was comparable (*p* = NS) in control and CsA-treated hearts (Figures 2D,E).

### Paradoxical effect of CsA

**Figure 3:**
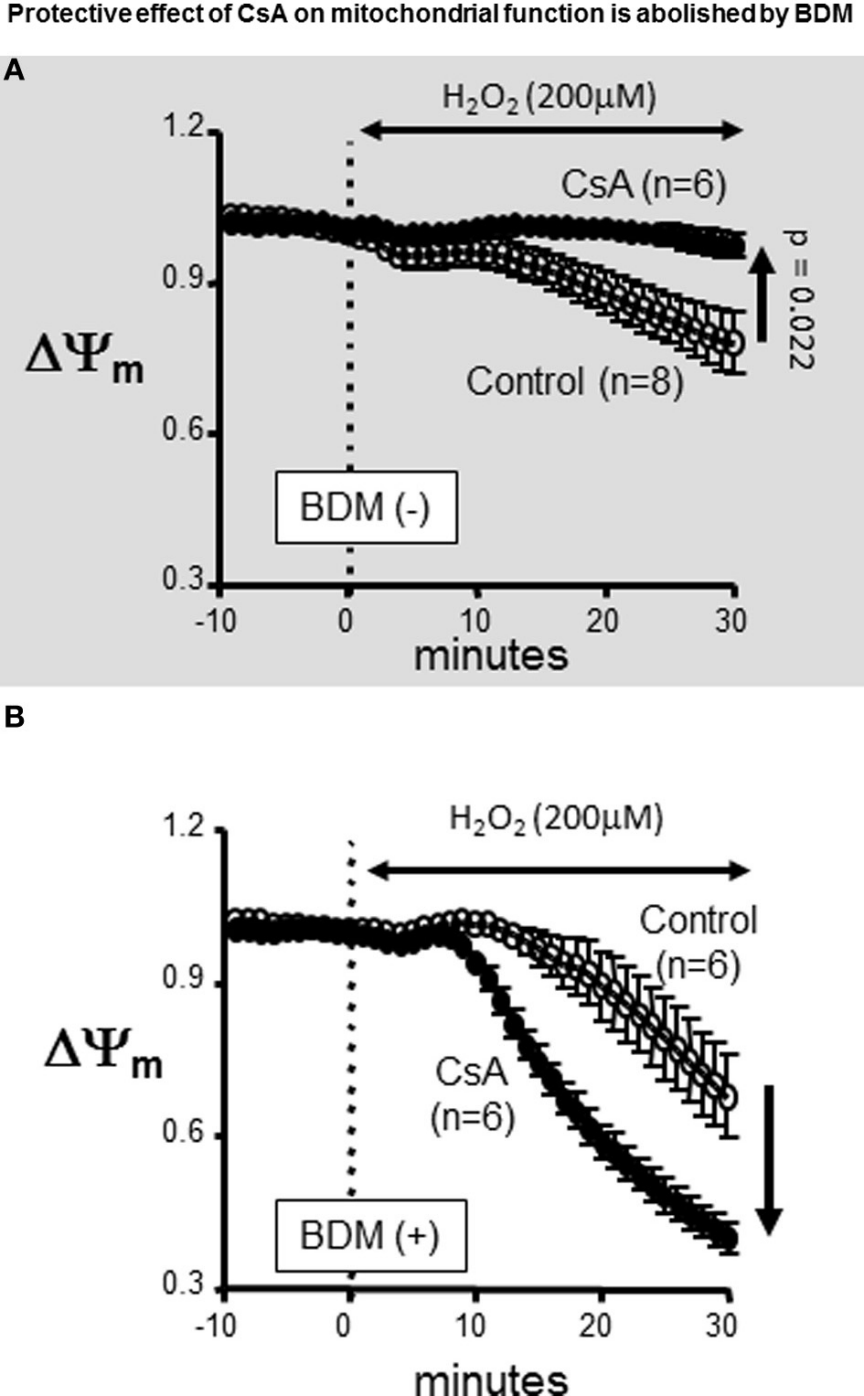
**Protective effect of CsA on mitochondrial function is abolished by BDM**. Treatment with the chemical phosphatase BDM abolishes the protective effects of CsA. **(A)** For reference, ΔΨ_m_ response presented in Figure 1 indicating protection by CsA against OS-induced ΔΨ_m_ depolarization. **(B)** Addition of the chemical phosphatase BDM (10 mM) reversed the effect of CsA on ΔΨ_m_. BDM, 2,3-Butanedione monoxime.

Previously, we and others have highlighted the importance of maintaining ΔΨ_m_ polarization in the protection against OS-induced arrhythmias (Akar et al., 2005; Brown et al., 2010; Lyon et al., 2010). We, therefore, proceeded to investigate the basis for our discrepant findings regarding the role of CsA in protecting against OS-induced ΔΨ_m_ depolarization but not electrical dysfunction. We hypothesized that differences in experimental settings, particularly with regards to the use of the electromechanical uncoupling agent BDM in AP but not ΔΨ_m_ studies may underlie the discrepant outcomes that we observed. Therefore, we repeated our ΔΨ_m_ measurements with and without addition of BDM to the perfusate. Remarkably, we found that BDM completely reversed the protective effect of CsA on OS-induced ΔΨ_m_ depolarization which we had initially observed (Figure 3A, gray background), as CsA-treated hearts exhibited a more rapid ΔΨ_m_ depolarization compared to untreated controls following H_2_O_2_ challenge when BDM was present in the perfusate (Figure 3). As such, the use of BDM revealed a paradoxical effect of CsA which was consistent with exacerbation rather than protection against OS-induced mitochondrial dysfunction. Importantly, BDM alone (i.e., without CsA) did not alter the ΔΨ_m_ response of the heart to OS.

### Protein kinase C inhibition abrogates the protective effect of CsA on OS-induced mitochondrial dysfunction

**Figure 4:**
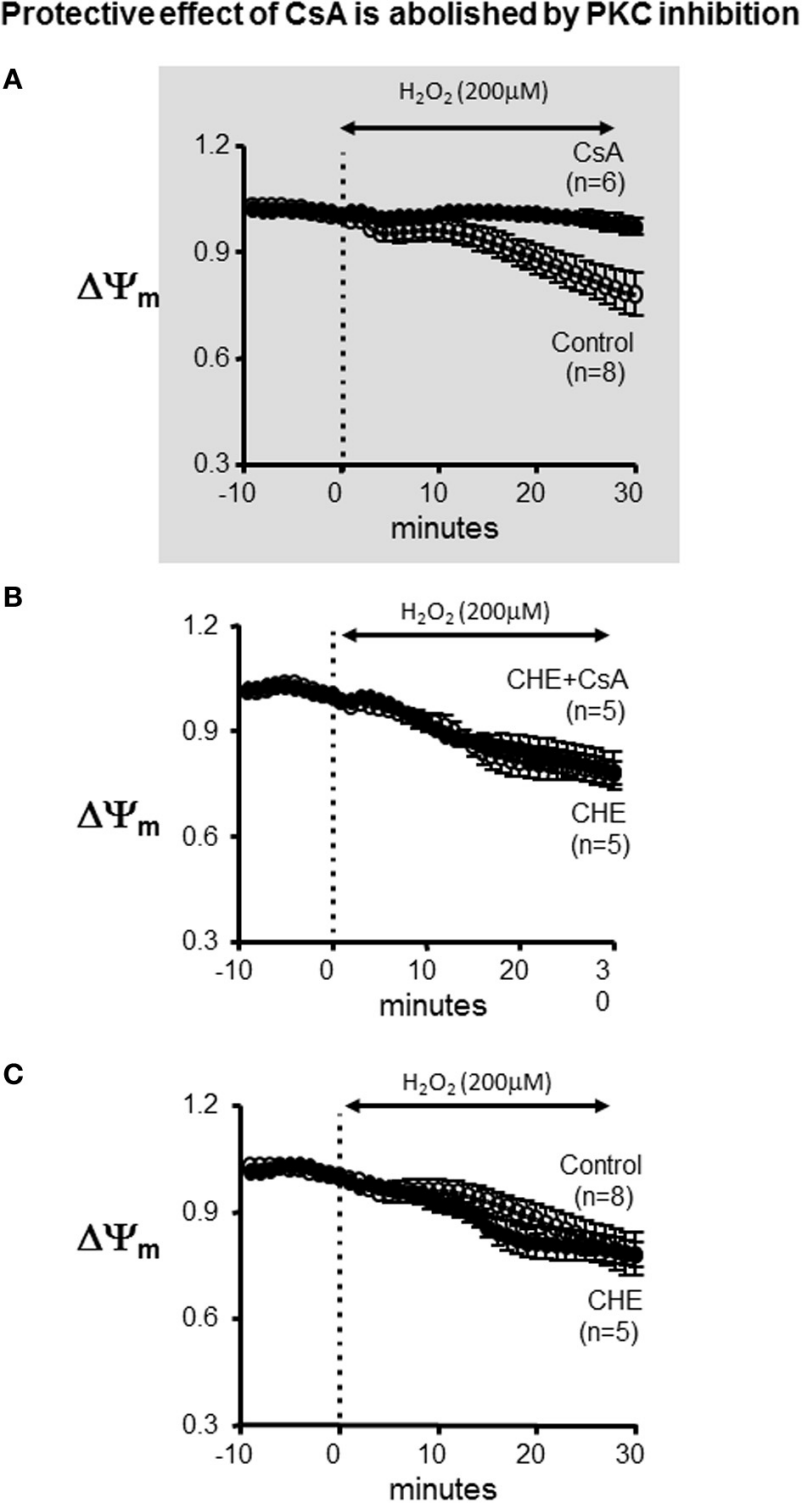
**Protective effect of CsA is abolished by PKC inhibition. (A)** For reference, ΔΨ_m_ response presented in Figure 1 indicating protection by CsA against OS-induced ΔΨ_m_ depolarization. **(B)** Average ΔΨ_m_ response to CsA treatment in the presence of the specific PKC inhibitor CHE. **(C)** Average ΔΨ_m_ response in control and CHE treated hearts. CHE alone (i.e., without CsA) did not alter the response. CHE, Chelerythrine; PKC, Protein kinase C.

BDM is a strong chemical phosphatase, which is known to oppose the phosphorylation of serine/threonine target proteins and to increase ATP depletion in metabolically challenged cardiomyocytes (Stapleton et al., 1998). Therefore, we hypothesized that the paradoxical effect that was unmasked by BDM in terms of CsA-mediated dysfunction may be due to its interference with the activity of the cardioprotective mK_ATP_ channel which is known to modulate mPTP opening at least *in vitro*. Since mK_ATP_ activity is dependent on PKC-mediated phosphorylation, we replaced BDM with the selective PKC inhibitor, CHE. As shown in Figure 4, addition of CHE completely abolished the protective effect conferred by CsA. Of note, CHE failed to alter the ΔΨ_m_ response of the heart to OS when delivered alone (i.e., without CsA) (Figure 4, lower panel).

### Direct mK_ATP_ blockade abrogates the protective effect of CsA on mitochondrial dysfunction

**Figure 5:**
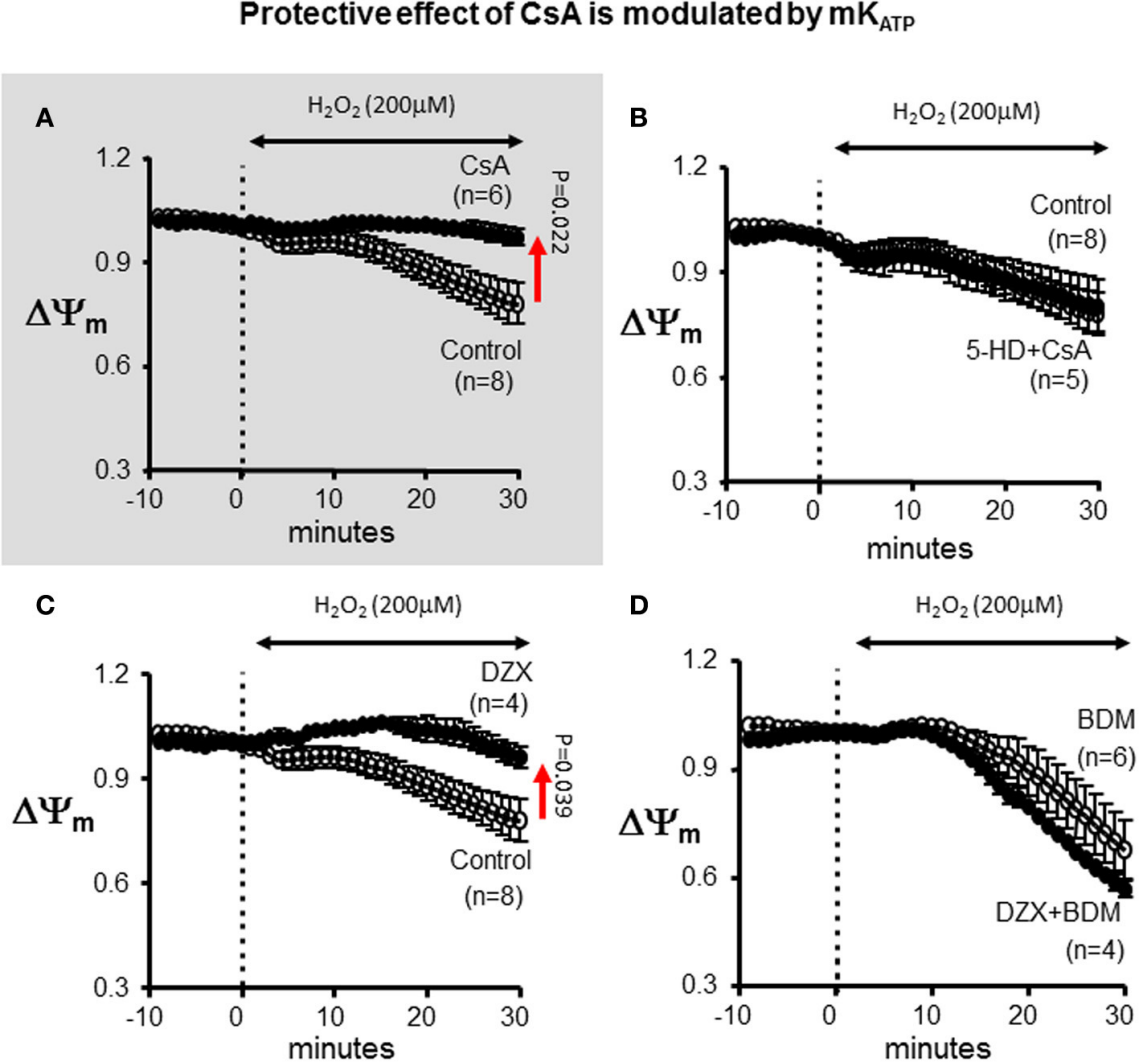
**Protective effect of CsA is modulated by mK_ATP_. (A)** For reference, ΔΨ_m_ response presented in Figure 1 indicating protection by CsA against OS-induced ΔΨ_m_ depolarization. **(B)** Treatment with the mK_ATP_ antagonist abrogates the protective effect of CsA on mitochondrial dysfunction. **(C)** mK_ATP_ activation by DZX protects against OS-induced ΔΨ_m_ depolarization. **(D)** BDM abolishes the protective effect of DZX on mitochondrial dysfunction. DZX, Diazoxide.

Since PKC has multiple mitochondrial targets that may alter the response of the heart to OS, we tested whether direct pharmacological blockade of mK_ATP_ recapitulated the inhibitory effects of CHE on CSA-mediated cardioprotection. Indeed, addition of 5-HD completely abrogated the protective effect of CsA on ΔΨ_m_ depolarization (Figure 5). The ΔΨ_m_ response to H_2_O_2_ was identical between the control and the combined 5-HD+CsA treated hearts, highlighting the notion that CsA was completely ineffective as a cardioprotective agent under conditions that prevented mK_ATP_ channel activation.

### Pharmacological activation of mK_ATP_ protects against mitochondrial dysfunction

To further establish the role of mK_ATP_ in the modulation of the ΔΨ_m_ response to OS, we used the pharmacological agonist of the channel, DZX (Figure 5). Interestingly, DZX-treated hearts exhibited a markedly blunted ΔΨ_m_ response compared to control hearts; thereby, establishing the efficacy of mK_ATP_ in modulating the opening of mPTP. Once again, the protective effect of DZX on OS-induced ΔΨ_m_ depolarization was prevented by addition of the chemical phosphatase BDM.

### Protective effect of CsA on arrhythmias is dependent on mK_ATP_ channel activation

**Figure 6:**
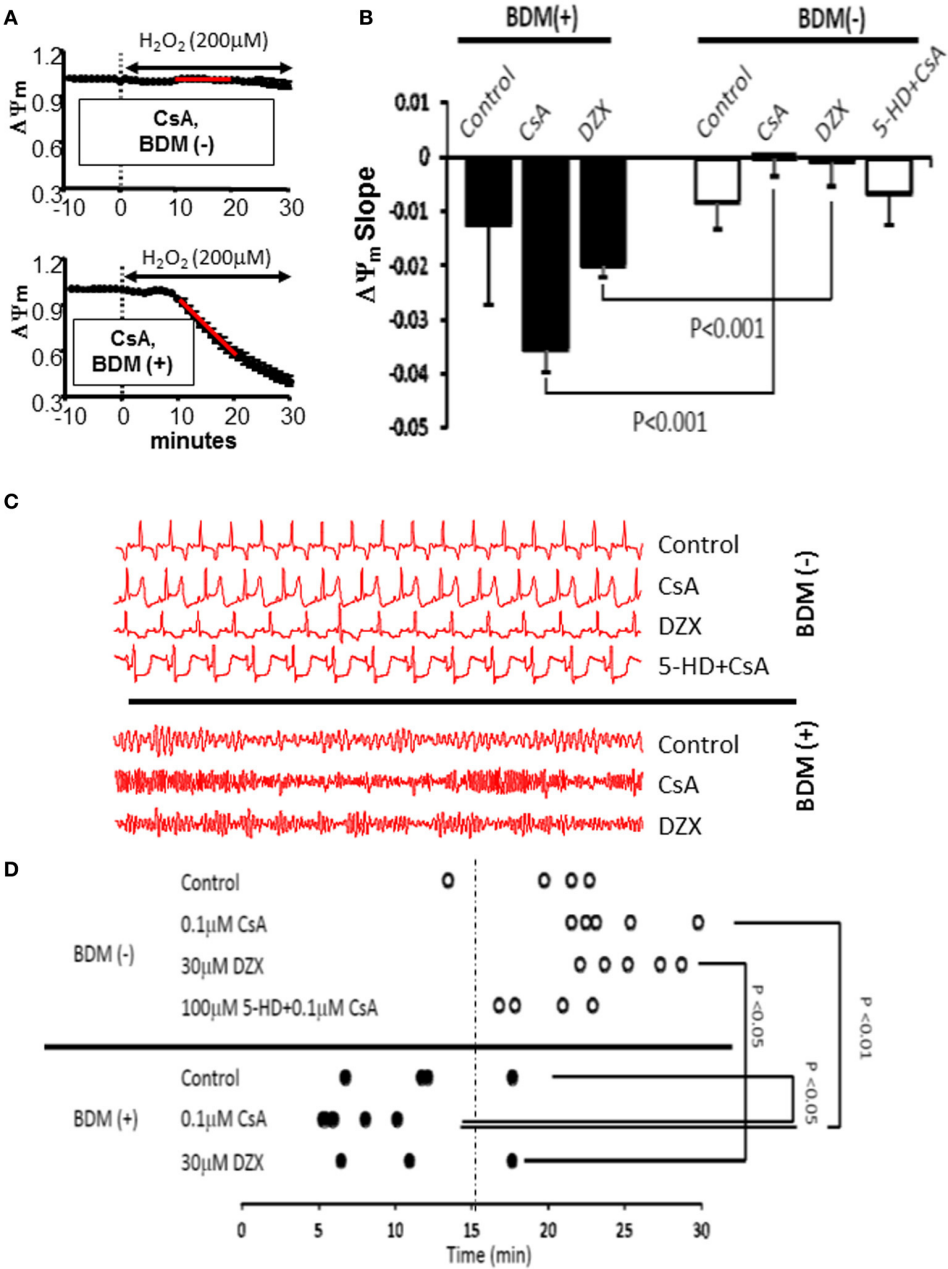
**Cross-talk between mK_ATP_and mPTP modulates mitochondrial and electrical response of hearts to OS. (A)** ΔΨ_m_ response to OS in CsA-treated hearts without (top) and with (bottom) concomitant perfusion with the chemical phosphatase BDM. The relative sensitivity of hearts to OS-induced mitochondrial dysfunction was quantified by measuring the slope of ΔΨ_m_ collapse 10–20 min following H_2_O_2_ perfusion (red line). **(B)** Average slope of OS-induced ΔΨ_m_ change in all groups tested. **(C)** Representative ECG traces from all groups tested, indicating vulnerability to VF in BDM (+) hearts. **(D)** Time to onset of VF following OS challenge as an index of electrical vulnerability in all hearts from all groups. VF, ventricular fibrillation.

Previously, we and others showed that interventions that stabilized ΔΨ_m_ were associated with protection against post-ischemic arrhythmias, whereas conditions leading to ΔΨ_m_ instability promoted electrical dysfunction (Akar et al., 2005). Therefore, we asked whether modulation of the ΔΨ_m_ response in this model of acute OS could also explain differential vulnerability to arrhythmias. Seven groups were examined in terms of their relative sensitivities to OS-induced mitochondrial dysfunction (quantified by the slope of ΔΨ_m_ depolarization) and electrical dysfunction (assessed by the time to onset of VF). As shown in Figure 6, conditions that led to accelerated ΔΨ_m_ depolarization were indeed associated with enhanced vulnerability to VF as they exhibited significantly (*p* < 0.05) shorter time to onset of VF in response to OS challenge. While 11/13 BDM (+) hearts exhibited early (within 15 min) onset of VF, only 1/19 BDM (−) hearts were prone to VF within this short time-frame. These findings indicate significantly heightened sensitivity to sustained arrhythmias of hearts treated with the chemical phosphatase (*p* = 0.000006).

**Figure 7:**
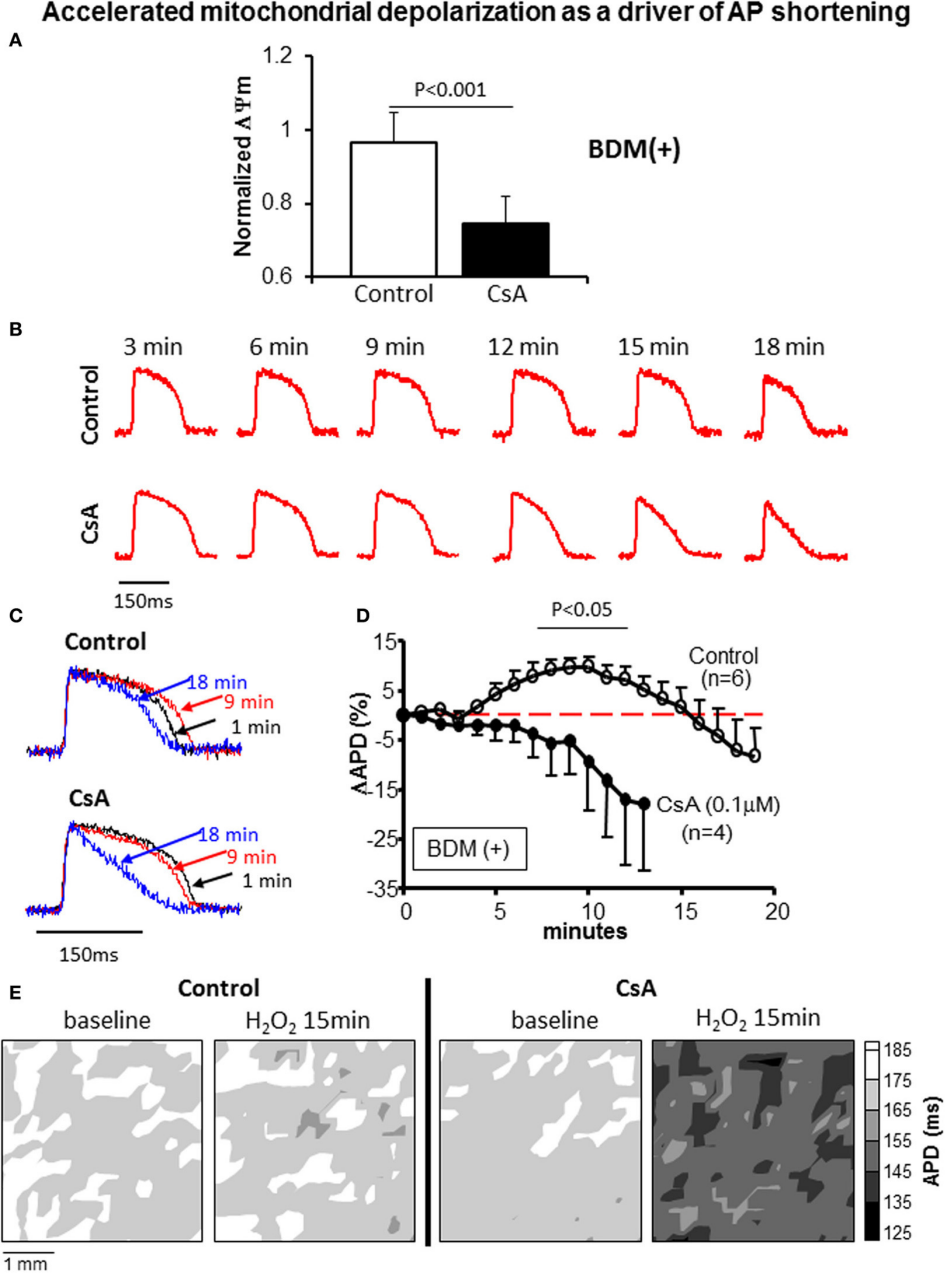
**(A)** ΔΨ_m_ at 15 min of OS normalized to baseline value in control and CsA treated hearts. **(B,C)** Representative action potentials recorded during early challenge with acute OS in control and CsA treated hearts. AP shortening is more pronounced in CsA compared to control hearts, indicating heightened sensitivity to OS. **(D)** OS-induced % change in APD relative to baseline (pre-H_2_O_2_ value) in control and CsA treated hearts. **(E)** Representative APD contour maps showing global shortening of APD in response to OS in CsA treated hearts compared to controls.

To further address the link between mitochondrial and electrophysiological instability and to elucidate the potential mechanism underlying the CsA mediated pro-arrhythmic effect which we uncovered under conditions that prevented mK_ATP_ channel activation and that led to more pronounced ΔΨ_m_ depolarization (Figure 7A), we performed detailed optical AP mapping. Analysis of AP properties revealed accelerated shortening of APD in response to OS in CsA-treated hearts compared to controls (Figures 7B–E). These data suggest a heightened electrophysiological sensitivity to OS, particularly with regards to the activation of repolarizing currents as the potential mechanism for CsA-mediated pro-arrhythmia (Figure 7).

## Discussion

Acute OS manifests in a majority of patients with coronary artery disease, the leading cause of arrhythmic deaths in the United States. Central to the pathology of OS is mitochondrial dysfunction. Although the role of mitochondria as mediators of cell injury is well established, their contribution to arrhythmias is less understood. Indeed, the exact mitochondrial transport pathways that modulate the susceptibility of the heart to electrical dysfunction remain unclear.

In the present work we focused on the mPTP because of its established role in cellular necrosis and MI. Specifically, we investigated the efficacy of CsA in protecting against OS-induced mitochondrial and electrical dysfunction. We chose a simple *ex vivo* model of H_2_O_2_ challenge which reliably and predictably causes ΔΨ_m_ depolarization and VF within a relatively short (<30 min) time-frame. Our experiments revealed important discrepancies which initially appeared to discredit our central hypothesis that stabilization of ΔΨ_m_ is anti-arrhythmic, as CsA seemed to protect against ΔΨ_m_ depolarization but worsen electrical dysfunction. Further analysis revealed the basis of these discrepant observations, as we uncovered a dual role for CsA in either protecting or impairing cardiac function depending on the cellular milieu. As will be discussed below, our initial findings led us to refine our central hypothesis by examining the functional cross-talk between the mPTP and the cardioprotective mK_ATP_ channels in ultimately mediating the response of the heart to OS. In the present work, we highlight the importance of mK_ATP_ channel availability in determining the efficacy of CsA as a cardioprotective agent.

### Mitochondrial ion channels as mediators of cardiac dysfunction: role of mPTP

ΔΨ_m_ is a key metric of mitochondrial function as it forms the proton-motive force used for ATP synthesis. In normal hearts, ΔΨ_m_ is tightly regulated such that ATP synthesis and ROS generation are maintained within a physiological range. In response to OS, ΔΨ_m_ is disrupted, altering over-all energy and redox balance within cardiac myocytes. Specifically, under these conditions, ROS build-up can exceed a threshold level that triggers the sequential opening of mitochondrial channels in a hierarchal manner (IMAC followed by mPTP) (Aon et al., 2007), which in turn leads to ΔΨ_m_ instability. ΔΨ_m_ instability can lead to inexcitability at the cellular level and conduction block and arrhythmias at the organ level, via a mechanism termed “*metabolic sink”* (Akar et al., 2005). Furthermore, pharmacological blockade of IMAC which blunted ΔΨ_m_ depolarization improved electrical and functional recovery of the heart following IR injury (Akar et al., 2005; Brown et al., 2008; Aon et al., 2009). That work, however, focused on relatively mild levels of OS produced by short episodes of IR injury. Since energy dissipating mitochondrial channels exhibit a hierarchal activation pattern in response to rising ROS levels (Aon et al., 2007), we focused in the present work on the efficacy of mPTP blockade by CsA in a model that was tailored to reliably depolarize ΔΨ_m_ and generate VF via MPT formation.

### Cyclosporin a as a cardioprotective agent

The initial success of the immunosuppressive agent CsA in reducing infarct size in patients with coronary artery disease through its potent CycP-D inhibitory activity has fueled considerable interest in its potential use as a therapeutic agent for a wide variety of cardiovascular disorders (Piot et al., 2008; Hausenloy et al., 2012). Despite these encouraging clinical findings, the efficacy of CsA in preventing arrhythmias is unclear (Arteaga et al., 1992; Ko et al., 1997; Schreiner et al., 2004), and recent experimental, preclinical (Lie et al., 2008), and clinical findings (Ghaffari et al., 2013) have cast new doubts regarding the overall utility of CsA. Our current work was designed to directly address issues related to CsA efficacy in improving metabolo-electrical function under conditions of OS.

Consistent with the expected therapeutic benefit of preventing irreversible mPTP opening, CsA treatment in our experiments significantly delayed the onset of OS-induced ΔΨ_m_ collapse and the loss of contractile function in *ex vivo* perfused hearts (Figures 1, 2). However, we found that this metabolo-contractile improvement did not translate into an electrical benefit (Figure 2D). Rather, we found evidence of compromised electrical function with no improvement in the onset of VF. As such, our findings regarding lack of arrhythmic protection are fully consistent with those of Artega et al. who reported impairment rather than protection against reperfusion arrhythmias (Arteaga et al., 1992).

Our overarching hypothesis is that stabilization of ΔΨ_m_ is anti-arrhythmic. However, our initial findings regarding improved mitochondrial but not electrophysiological function by CsA appeared to disprove this premise. This prompted us to examine this issue in greater detail. As will be discussed next, our subsequent experiments led to the discovery of an intricate cross-talk within a mitochondrial macromolecular complex that ultimately dictated the functional response of the heart to OS, and conferred upon CsA a dual role as a mediator of protection or dysfunction depending on the specific cellular milieu.

### Cross-talk between mPTP and mK_ATP_ in modulating ΔΨ_m_ and arrhythmias

A major finding of the present report is the demonstration that the cardiac response to OS is dictated by complex cross-talk between multiple mitochondrial transport mechanisms. Indeed, we found that OS is mediated through an intra-mitochondrial signaling pathway that can either worsen or protect against arrhythmias depending on the nature of its activation. We first showed that BDM, a classical electromechanical uncoupling agent, not only abrogated the protective effect afforded by CsA but rather led to an acceleration of OS-induced mitochondrial depolarization in response to CsA treatment. This paradoxical effect can explain the worsened electrical outcome in terms of APD shortening that we saw upon CsA treatment. Indeed, the synergy between accelerated mitochondrial depolarization and APD shortening is fully consistent with previous cellular and organ level findings (Akar et al., 2005; Aon et al., 2007). It is important to note that BDM alone (without CsA) did not alter the mitochondrial response to OS. This highlights an interaction of BDM with key proteins that modulate the mPTP (the main target of CsA).

Since BDM inhibits phosphokinases and actively dephosphorylates serine/threonine residues on multiple proteins (Stapleton et al., 1998), we hypothesized that the phosphorylation state of a certain target protein which interacts with elements of the mPTP may be critical for mediating the protective effects of CsA. Given the established role of PKC in mediating cardioprotection by ischemic pre and post-conditioning (Inagaki et al., 2006), we tested whether the detrimental effects of BDM could be explained (at least partially) through its PKC inhibitory activity. We addressed this issue by replacing BDM with the specific PKC inhibitor, CHE. Here too, protection against OS-induced mitochondrial dysfunction by CsA was completely abolished. As such, our findings are consistent with elegant work by the Mochly-Rosen group who highlighted the importance of PKC mediated signaling in the protection against mitochondrial dysfunction in multiple organ systems, including the heart, as well as pioneering molecular and biochemical work from the Garlid laboratory demonstrating the desensitization of mPTP to ROS by PKCε. Unlike BDM, however, CHE did not accelerate (i.e., worsen) the rate of mitochondrial depolarization suggesting involvement of additional off target effects by BDM that adversely impact mitochondrial function. These may include the effects of BDM on a variety of tyrosine kinases as well as its reported role in depleting ATP levels in myocytes under conditions of metabolic challenge (Stapleton et al., 1998). Our present findings not only inform regarding the signaling pathways involved in CsA mediated cardioprotection but also serve to emphasize the need to exercise caution when interpreting findings of studies in which BDM is used, especially those addressing issues relating to metabolic stress.

PKC has numerous target substrates that can impact mitochondrial function either directly or indirectly. One critical target of PKC signaling which has emerged from elegant work by the Marban group and others is the mK_ATP_ channel (Sato et al., 1998). We tested whether direct pharmacological modulation of the channel could potentially explain the detrimental effects of PKC inhibition which we observed. Indeed, we found that 5-HD was as effective as CHE in fully abrogating the protective effects of CsA on OS-induced ΔΨ_m_ depolarization. Although Baines et al. demonstrated that PKCε interacts with multiple key components of the mPTP, including VDAC, ANT, and HKII independently of its interaction with mK_ATP_(Baines et al., 2003), our present work argues that such mK_ATP_-independent interactions do not impact the functional response of the intact heart to OS. Indeed, we provide functional data that extend previous *in vitro* studies and give credence to the notion that mK_ATP_ is the central mediator of the cardioprotective effects of PKC on the heart. In light of the importance of mK_ATP_ we went on to investigate the functional consequences of channel activation and found that DZX treatment was as protective as CsA in preventing ΔΨ_m_ collapse. Once again, that protection was prevented by the chemical phosphatase BDM, consistent with the notion that PKC mediated phosphorylation of mK_ATP_ is critical to its opening and therefore efficacy in cardioprotection.

## Limitations

Our study has several important limitations that require mention. For one, we relied upon a pharmacological strategy using DZX and 5-HD to modulate the activity of mK_ATP_. Although this standard approach which included carefully chosen concentrations was based on numerous published reports, we cannot fully exclude the possibility that minor mK_ATP_-independent effects may have contributed to our findings.

Moreover, we used a non-ratiometric, semi-quantitative method of TMRM imaging to assess relative (not absolute) changes in mitochondrial function in *ex vivo* perfused hearts. Using this validated method, changes in TMRM fluorescence signal caused by altered cellular membrane potential as opposed to ΔΨ_m_ are negligible.

Finally, we used CsA to inhibit the mPTP. While this is a widely accepted and effective strategy in the heart, Li et al. have shown tissue-specific differences in CyP-D expression and therefore sensitivity to CsA (Li et al., 2012). In particular, they reported that mPTP inhibition in tissues exhibiting low expression of CyP-D, is achieved more effectively using Rotenone than CsA (Li et al., 2012).

## Conclusion

In summary, our current findings highlight the notion that CsA-mediated cardioprotection against OS requires mK_ATP_ channel activation through a PKC-dependent pathway. Increasing mK_ATP_ activity during CsA administration is required for limiting OS-induced electrical dysfunction. On the other hand, CsA administration during conditions that may prevent mK_ATP_ channel activation may exert unintended pro-arrhythmic consequences through accelerated APD shortening. Our findings may explain existing controversy in the basic and clinical literature surrounding the utility of CsA as a cardioprotective agent.

### Conflict of interest statement

The Associate Editor Miguel A. Aon declares that, despite publishing articles in the past with author(s) Fadi G. Akar, Chaoqin Xie, and Justin Kauffman, the review process was handled objectively and no conflict of interest exists. The authors declare that the research was conducted in the absence of any commercial or financial relationships that could be construed as a potential conflict of interest.
